# Le distichiasis: une anomalie des cils

**DOI:** 10.11604/pamj.2015.20.55.6044

**Published:** 2015-01-21

**Authors:** Seydou Bakayoko, Nouhoum Guirou

**Affiliations:** 1 Centre Hospitalier Universitaire - Institut d'Ophtalmologie Tropicale d'Afrique Boulevard du Peuple, Bamako, Mali

**Keywords:** Distichiasis, glandes de Meibomius, lymphœdème-distichiasis, distichiasis, meibomian glands, lymphedema-distichiasis

## Image en medicine

Le distichiasis est une anomalie congénitale rare à transmission autosomique dominante dans laquelle il existe une deuxième rangée anormale de cils, localisée au niveau des glandes de Meibomius. Cette seconde rangée de cils est très souvent en contact avec le globe oculaire entrainant des complications cornéennes. Le gène en cause FOXC2 (Forkhead Box C2) localisé en position 16q24.3, participe à l'embryogenèse du système lymphatique et vasculaire, d'où l'appellation de lymphœdème-distichiasis. En dehors du lymphœdème, d'autres anomalies cardiovasculaires et ORL peuvent être associées.

**Figure 1:**
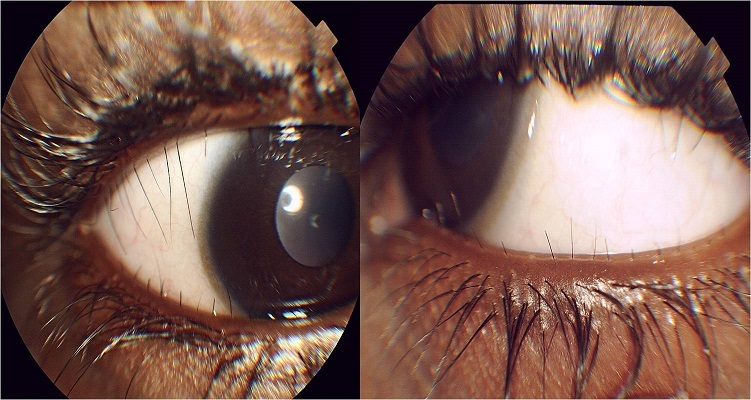
Œil droit et gauche d'un patient montrant une seconde rangée de cils

